# Impact Assessment of Lead-Tolerant Rhizobacteria to Improve Soil Health Using Indian Mustard (*Brassica juncea*) as an Indicator Plant

**DOI:** 10.3390/plants12163005

**Published:** 2023-08-21

**Authors:** Zain Mushtaq, Adnan Akhter, Hafiz Azhar Ali Khan, Waheed Anwar, Abeer Hashem, Graciela Dolores Avila-Quezada, Elsayed Fathi Abd_Allah

**Affiliations:** 1 Department of Soil Science, Faculty of Agricultural Sciences, University of the Punjab, Lahore P.O. Box 54590, Pakistan; 2 Department of Plant Pathology, Faculty of Agricultural Sciences, University of the Punjab, Lahore P.O. Box 54590, Pakistanwaheedanwar.dpp@pu.edu.pk (W.A.); 3 Department of Entomology, Faculty of Agricultural Sciences, University of the Punjab, Lahore P.O. Box 54590, Pakistan; azhar.iags@pu.edu.pk; 4 Botany and Microbiology Department, College of Science, King Saud University, P.O. Box 2460, Riyadh 11451, Saudi Arabia; habeer@ksu.edu.sa; 5 Facultad de Ciencias Agrotecnológicas, Universidad Autónoma de Chihuahua, Chihuahua 31350, Mexico; gdavila@uach.mx; 6 Plant Production Department, College of Food and Agricultural Sciences, King Saud University, P.O. Box 2460, Riyadh 11451, Saudi Arabia

**Keywords:** heavy metal, Indian mustard, rhizobacteria, metal contamination

## Abstract

Due to ongoing human activities, heavy metals are heavily accumulated in the soil. This leads to an increase in the discharge and the quick spread of heavy metal pollution in human settlements and natural habitats, having a disastrous effect on agricultural products. The current experiment was planned to evaluate the effect of lead-tolerant-plant-growth-promoting rhizobacteria (LTPGPR) on growth, yield, antioxidant activities, physiology, and lead uptake in the root, shoot, and seed of Indian mustard (*Brassica juncea*) in lead-amended soil. Three pre-isolated well-characterized lead-tolerant rhizobacterial strains—S10, S5, and S2—were used to inoculate seeds of Indian mustard grown at three different levels of lead (300 mg kg^−1^, 600 mg kg^−1^, 900 mg kg^−1^) contaminated soil. The experiment was designed following a completely randomized design (CRD) under factorial arrangements. Lead nitrate was used as a source of lead contamination. At harvesting, data regarding growth, physiology, yield per plant, antioxidant activities, malondialdehyde and proline content, and lead uptake in the root, shoot, and seed of Indian mustard were recorded. Results demonstrated that lead contamination at all levels significantly reduced the plant growth, yield, and physiological processes. Plants inoculated with lead-tolerant rhizobacteria showed a significant improvement in plant growth, yield, antioxidant activities, and physiological attributes and cause a valuable reduction in the malondialdehyde contents of Indian mustard in lead-contaminated soil. Moreover, plants inoculated with lead-tolerant rhizobacteria also showed an increment in lead uptake in the vegetative parts and a significant reduction of lead contents in the seed of Indian mustard.

## 1. Introduction

Soil is an essential natural resource that plays a crucial role in agricultural sustainability. However, the issue of soil contamination caused by a variety of contaminants, both organic and inorganic, has garnered worldwide concern due to the significant risks soil contamination poses to the environment and human health. The presence of heavy metal (HM) contamination in soil poses a significant threat to both soil health and plant productivity, as well as resulting in irreversible harm to humans through their incorporation into the food chain [1]. Hazardous materials (HMs) exhibit non-biodegradability, meaning that they remain in soils for extended periods of time and can be transported to remote locations due to their heightened solubility in water [1,2]. The dissemination of pollutants in the environment can be attributed to a range of sources, including both natural and anthropogenic sources. Natural sources of heavy metals include the transport of continental dust and atmospheric emissions. On the other hand, human activities such as mine exploitation, the application of sewage water for irrigation, the use of agrochemicals enriched with metals, and the smelting of metals from ores contribute to the spread of heavy metals.

The cultivation of agricultural sustainability is entirely dependent on soil, it being a valuable natural resource [2]. Soil metal contamination through anthropogenic activities is a major global concern and threat to mankind. Pollution is one of the phenomenal offshoots witnessed in this evolutionary world. It refers to the contamination of the air, soil, and water bodies as a result of the mixing of undesired substances resulting from various activities, including those stemming from the industrial sector, natural processes, calamities, urbanization, and waste treatment plants. Industrialization and urbanization are most important for the prosperity of the national economy, both being major sources of the development of a country, but there are certain drawbacks that, side by side, disturb the overall process. Increased industrialization, however, is needed to meet the requirements of today’s life in terms of both economic and social development; this is despite the major negative impact of industries that pollute the environment, ultimately affecting human health [1]. One of the big threats to the environment is pollution caused by heavy metals due to increased industrialization. The chemical industries are polluting the soil, air, and water and, most importantly, are disturbing the natural process. The most important feature relating to heavy metals is the fact that they are non-biodegradable and remain in the system, requiring manual deactivation [3].

Lead (Pb) is a poisonous, toxic, and persistent heavy metal [4]. Lead toxicity in soil, air, and water has increased significantly due to anthropogenic activity. Its contamination might have been increased due to the weathering of rocks, the paint industries, the use of sewage-water for agriculture, and the increased use of leaded gasoline in nuclear industries [5]. According to the World Health Organization (WHO), the Pb concentration in most of the regions of Pakistan has increased to a critical level [6].

Phytoremediation is an environmentally friendly decontamination mechanism moderated by the crop plants [7]. Trees, shrubs, and grasses, in relationship with microbes, mediate the polluted environment [8]. The metal uptake capabilities of plants are very important to this technique [9]. Phytoremediation is a vital green technology with the potential to remove contaminants from the soil (organic pollutants, heavy metals, etc.) while reducing the production of secondary waste [10]. It is environmentally friendly, cost-effective, operationally feasible, and relatively simple technology. Most importantly, phytoremediation is easy to implement as it does not require expensive equipment or expert personnel [11]. However, phytoremediation is a slow process; it might take several years for the restoration of metal-contaminated soils to a standard, healthy state. Conditions may change depending upon the sensitivity of metals and soil type [10].

Phytoremediation efficacy can be improved through the interaction of metal-tolerant-plant-growth-promoting rhizobacteria, which ameliorate plant growth and development under metal stress environments through different direct and indirect mechanisms [12]. These mechanisms include the phytohormonal production of cytokinin’s, gibberellic acid or indole acetic acid, ACC production under stress condition, mineral solubilization such as via phosphorous and potassium, the production of antifungal metabolites, and, most importantly, the immobilization of heavy metals through exopolysaccharides (EPS) production [4,6]. Agricultural scientists have explained that metal-resistant microbial inoculation could improve plant health and survival under a polluted environment due to the production of growth regulators such as cytokinin, auxin, gibberellins, the enzymatic lowering of ethylene, etc. [13,14]. Plant–microbe interaction is an efficient and successful bioremediation process for contaminant degradation, metal bioaccumulation, and for improving plant growth.

*Brassica juncea*, commonly known as Indian mustard, is widely recognized as a proficient accumulator of lead (Pb) due to its rapid growth and substantial biomass generation [15]. Consequently, it has found extensive application in the domain of heavy metal pollution remediation. Furthermore, *B. juncea* exhibits the capability to mitigate heavy metal pollution, and it also possesses the ability to eliminate organic pollutants from the surrounding environment via various mechanisms such as uptake, rhizodegradation, and other pathways. To date, there exists a limited number of studies documenting the utilization of *B. juncea* for the purpose of mitigating the presence of heavy metals and pesticides, particularly in the context of remediating soil contaminated by industrial effluents [16].

Based on current available information, it appears that there is a lack of research conducted on the utilization of lead-tolerant rhizobacteria for the purpose of phytoremediation in soil that has been consistently exposed to industrial effluents. This study presents the development of a novel remediation strategy that combines plants and microorganisms to address soil pollution caused by lead (Pb). Specifically, the strategy involves cultivating Indian mustard plants and inoculating them with plant-growth-promoting rhizobacteria (PGPR). This study has the potential to offer an environmentally friendly and sustainable method for decontaminating soil polluted with lead (Pb) through the implementation of improved phytoremediation techniques. The goal of the current experiment was to assess the influence of lead-tolerant-plan- growth-promoting rhizobacteria on growth, yield, physiological characteristics, antioxidant activities, and lead uptake in Indian mustard.

## 2. Results

### 2.1. Agronomic Growth Parameters

The shoot length of Indian mustard was significantly reduced in Pb-contaminated soil. The increase in Pb contents severely reduced the shoot length. Lead contamination at 900 mg kg^−1^ significantly reduced the shoot length up to 40% compared with the plants grown in normal soil. However, the application of LTPGPR improved the shoot length in Pb-amended soil. Data depicted that inoculation with rhizobacterial strain S-10 significantly improved the shoot length up to 18.45% compared with control plants at the same level of contamination without inoculation (Table 1). The shoot fresh weight (SFW) was significantly reduced by Pb contamination at 900 mg kg^−1^. However, plants inoculated with S-5 exhibited a significant increase (15.98%) in shoot fresh weight compared with control plants. Shoot dry weight of Indian mustard was significantly reduced at all levels of lead. Maximum reduction (39.28%) was recorded at 900 mg kg^−1^ Pb contamination. However, inoculating the Indian mustard with bacterial strains significantly improved the shoot dry weight in Pb-contaminated soil. Inoculation with S-10 enhanced the shoot dry weight significantly up to 25%, compared with plants grown without inoculation at the same of level of Pb contamination (Table 1).

### 2.2. Lead Pollution Has a Substantial Negative Impact on Root Characteristics

Root length decreased up to 52.51% at 900 mg kg^−1^ lead contamination. However, S-5 inoculation improved the root length up to 31.77% at same level of contamination. The increase in Pb contents also reduced the root fresh and dry weight. Maximum reduction of 51 and 52.74%, respectively, in root fresh and dry weight was recorded at 900 mg kg^−1^. However, inoculating the plants with LTPGPR improved the root fresh and dry weight up to 40 and 50%, respectively.

Pod formation in Indian mustard was severely reduced through lead contamination. Pod numbers were reduced at all levels of contamination. Lead contamination at 900 mg kg^−1^ significantly decreased the number of pods up to 40.46% when compared with un-inoculated plants. However, plants inoculated with lead-tolerant bacteria significantly improved the number of pods. S-2 inoculation improved the number of pods up to 12% at 900 mg kg^−1^ lead contamination, compared with control plants grown without inoculation. The number of seeds per pod were significantly improved through inoculation. Inoculation with S-5 enhanced the number of seeds up to 10.20% at 900 mg kg^−1^, compared with plants grown at same level of contamination. Indian mustard yield was severely reduced in lead contamination. Lead contamination at 900 mg kg^−1^ decreased the yield up to 50% compared with control plants. Inoculation at levels improved the yield per plant significantly compared with un-inoculated plants. The Indian mustard yield per plant was enhanced up to 28.56% upon inoculation with S-5.

### 2.3. Chlorophyll a, b and Carotenoids Contents

However, Pb contamination significantly affected the chlorophyll a, b, and carotenoids of Indian mustard compared with un-inoculated control plants. Results revealed that Pb at 900 mg kg^−1^ caused a significant reduction of 29.34, 36.84, and 29.90%, respectively, in chlorophyll a, b, and carotenoids. Plants inoculated with LTPGPR enhanced the chlorophyll a, b, and carotenoids at all levels of contamination. Chlorophyll a and carotenoids contents improved up to 9.40 and 12.33%, respectively, through S-10 inoculation, and chlorophyll b increased up to 12% through S-2 inoculation at 900 mg kg^−1^ lead contamination compared with control plants (Table 2).

### 2.4. Estimation of Antioxidant Activity

Inoculation with LTPGPR improved the antioxidant activity of ascorbate peroxidase (APX), catalase, superoxide dismutase (SOD), proline, and glutathione reductase (GR), while causing a reduction in malondialdehyde (MDA) contents. Ascorbate peroxidase and catalase activity improved up to 20.41 and 18.11% through inoculation with S-5 and S-2, respectively, as compared with uninoculated plants grown in Pb contamination at 900 mg kg^−1^. An increment of 20.02% in superoxide dismutase (SOD) was recorded through inoculation (S-2) compared with uninoculated plants at 900 mg kg^−1^ spiked soil. Glutathione contents were improved up to 15.12% at 900 mg kg^−1^ through inoculating the plants. Bacterial inoculation also improved the proline contents of Indian mustard at all levels of metal contamination. However, it was found that Pb-tolerant bacteria caused a reduction of 38.48% in MDA contents in lead-amended soil as compared with plants grown in lead-contaminated soil without bacterial inoculation (Table 3). 

### 2.5. Lead Uptake in Vegetative and Reproductive Parts

**Figure 1:**
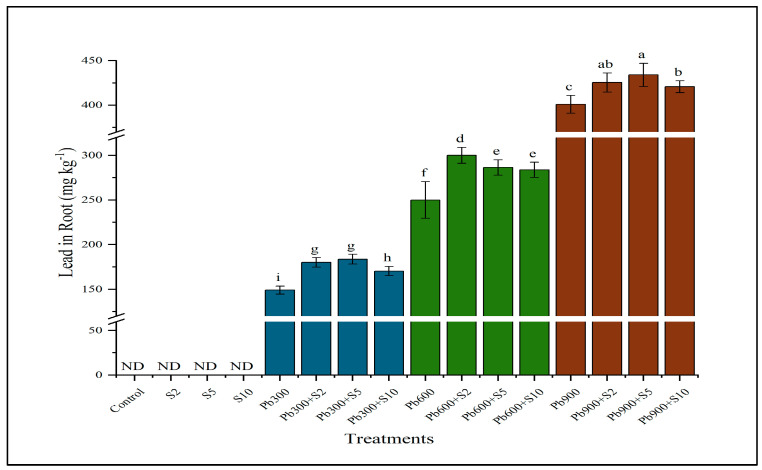
Result of lead−tolerant plant growth promoting rhizobacteria on lead (Pb) contents in roots. Means sharing same letter(s) don’t differ significantly at *p* < 0.05. ND refers to non-detectable values.

Data revealed that inoculation had a significant effect on Pb concentration in the root, shoot, and seed of Indian mustard. Inoculation with LTPGPR improved the lead uptake in root at all levels of contamination compared with un-inoculated plants at the same levels of contamination. At 900 mg kg^−1^, inoculation with lead-tolerant rhizobacteria (S2) ameliorated the lead uptake up to 9.2% compared with plants grown at the same level of contamination without inoculation (Figure 1).

**Figure 2:**
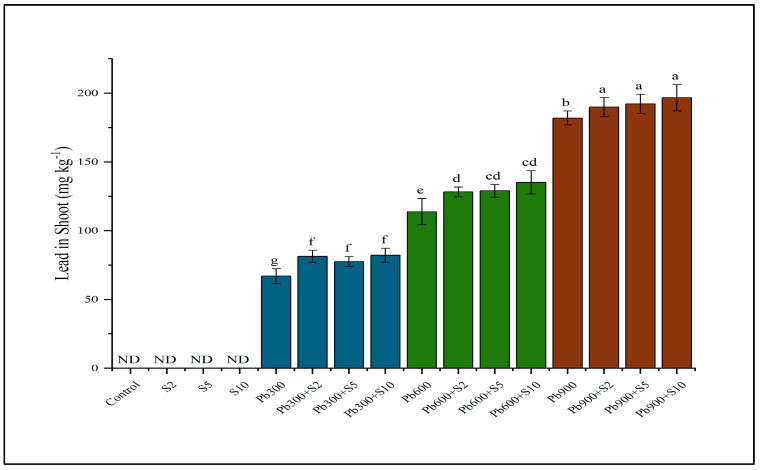
Result of lead−tolerant plant growth promoting rhizobacteria on lead (Pb) contents in shoots. Means sharing same letter(s) don’t differ significantly at *p* < 0.05. ND refers to non-detectable values.

Lead concentration in the shoot of Indian mustard was also improved through inoculation at different levels of Pb. Plants inoculated with rhizobacteria (S10) showed a significant increment of Pb uptake in the shoots of Indian mustard at 900 mg kg^−1^ spiked soil (Figure 2).

**Figure 3:**
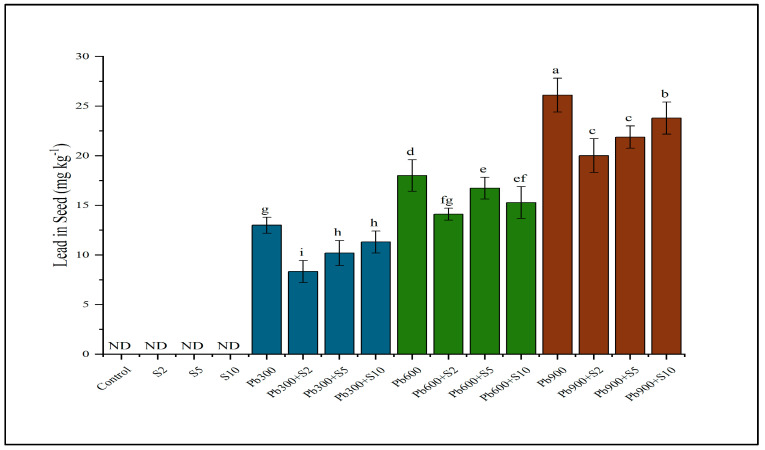
Result of lead−tolerant plant growth promoting rhizobacteria on lead (Pb) contents in seeds. Means sharing same letter(s) don’t differ significantly at *p* < 0.05. ND refers to non-detectable values.

Lead in the seeds of Indian mustard was significantly decreased through inoculating the plants with Pb-tolerant rhizobacteria. A significant reduction (26%) was recorded in seeds of plants inoculated with S5 at a contamination level of 900 mg kg^−1^ (Figure 3).

## 3. Discussion

Environmental threats due to increased metal pollution are of paramount concern. Increased industrialization, developmental activities, and ever-increasing urbanization are the main reasons behind metal pollution. Heavy metal concentration above the permissible limit has demonstrated detrimental impacts on soil, air, and water quality [17]. Anthropogenic activities have contributed significantly to metal pollution [18]. Nowadays, under an adverse environment, one of the serious issues for scientists is discovering possible means of improving plant health, the maintenance of plant productivity, and homeostasis [18].

Lead, one of the most common pollutants, is highly toxic [19]. The present study was executed in Pb-amended soil to find out the impact of lead contamination on physiological characteristic in the vegetative and reproductive parts of Indian mustard. Lead significantly reduced the growth, yield, antioxidant activity, and plant physiological processes in contaminated soil compared with natural soil without any metal contamination. The current work is in consensus with the findings of [20]. The reduction in growth, yield, antioxidant, and physiological processes might be due to the metal-induced reduction in the photosynthesis process, structural changes in the ultra-structure of chloroplast and the stomatal opening, disturbance in cell wall permeability, and, most importantly, inhibition of the electron transport chain reaction [21,22] The inhibition of root–shoot growth and the acceleration of peroxidation in leaves and roots are due to the presence of Pb. Lead also affects the enzyme activities that are involved in the Calvin cycle and nitrogen and sugar metabolism. The adverse effects of Pb may lead to the production of reactive oxygen species (ROS) (H_2_O_2_, OH, O_2_), which may result in oxidative stress [23]. This might be because Pb prevents iron inclusion in the photosynthesis process. Lead reduces chlorophyll production either by minimizing Fe and Mg uptake or by lowering chlorophyllase activity [24].

In the current study, inoculation with LTPGPR improved plant growth and physiology by reducing the negative effect of Pb. The significant improvement in plant growth and developmental processes through inoculation with metal-tolerant rhizobacteria might be due to the nutrient solubilization (phosphate, iron), siderophore, and phytohormone production along with significant systematic resistance against metal toxicity [25]. Several researchers explained that PGPR promotes the plant growth, development, physiological attributes, and yield under metal-contaminated soils [26]. It has been found that these metal-resistant bacteria also promote plant growth through synthesizing ACC-deaminase (1-aminocyclopropane-1-carboxylate), which cause a significant reduction in ethylene mediated stress [27]. Ref. [3] reported that inoculation with *Pseudomonas fluorescens* reduced the Pb toxicity in sunflower and significantly improved the plant growth under pot trial. Inoculation with Cr^6+^-reducing bacteria improved the grain yield up to 44% and caused a significant reduction (53%) in Cr^6+^ in the presence of compost. Previous studies explained that inoculation with rhizobacteria increased the phytoextraction efficiency, mainly by increasing the survival efficiency and yield of dry biomass on metal-contaminated soils. Improvement in plant physiological process through inoculation might be due to the fact that Pb-tolerant rhizobacteria increased the iron uptake, plausibly improved the chlorophyll production, and increased the photosynthetic activities [28].

In our study, inoculation improved the antioxidant activity (APX, SOD, GR) and proline contents in plants grown in leaded soil compared with control plants. To survive metal toxicity, plant species activate its antioxidant system to control metal detoxification. To counteract the harmful effect of reactive oxygen species (ROS), plants promote antioxidant activities that protect the plants against oxidative stress. An increase in malondialdehyde (MDA) contents in lead contamination is a signal of oxidative stress [29,30].

The bacterial inoculation improved the APX, SOD, GR, and proline content due to its enhancing of the antioxidant activity in lead-contaminated soil. The current experiment revealed that lead-tolerant rhizobacteria decreased the MDA contents in Indian mustard that might be due to the stimulatory effect of lead-tolerant bacteria on the plant defense system [31].

Lead-tolerant-plant-growth-promoting rhizobacteria resulted in higher accumulation of Pb content in the root and shoot of Indian mustard in Pb-amended soil compared with un-inoculated plants [32]. Improvement in Pb uptake might be due to the ability of rhizobacteria to decrease the soil pH that plays an important role in metal solubilization and uptake. Rhizobacteria could have produced chelates and organic acids and caused redox changes [33]. By changing the redox potential and solubility of Pb, plant-growth-promoting-rhizobacteria enhanced the metal uptake. Root exudates also play an important role in the current scenario. Root excretes the protons and organic acids that result in the acidification of soil, increased metal mobility, and decreased adsorption. These Pb-tolerant bacteria also improve phytoremediation through producing plant hormones [34]. Inoculation with Pb-tolerant bacteria reduced the Pb contents in the seeds of Indian mustard because of the metal immobilization and precipitation in the root and shoot by negatively charged particles. While this increased lead sequestration in the shoots, it decreased the lead translocation to the seeds of Indian mustard [35]. Lead-tolerant rhizobacteria improved the soil nutritive status through nitrogen fixation and nutrient solubilization, improved plant growth hormonal production, and protected the plant from metal stress through ACC-deaminase production [34,36].

## 4. Materials and Methods

A pot experiment was executed to evaluate the impact of pre-isolated well-characterized Pb-tolerant bacteria S10, S5, and S2 on plant growth and the development of Indian mustard in Pb-contaminated soil [37].

### 4.1. Seed Inoculation

Microbial inoculum was prepared in 250 mL LB media and incubated in the shaking incubator. The shaking incubator was set to 100 rpm at 28 ± 2 °C for 2–3 days. OD was measured to attain the 10^8^–10^9^ CFU mL−1 microbial population at 535 nm. The seeds were thoroughly surface sterilized by dipping in 0.2% HgCl_2_ and 95% ethanol for three minutes. For seed inoculation, peat-based slurry along with sugar solution (10%) as a sticky material was used and air-dried for 6–8 h.

### 4.2. Experiment-Setup

A pot trial was conducted with three well-characterized strains along with three different levels of lead (300, 600, 900 mg/kg). A total of 16 treatments were used along with 1 control treatment with different combinations of metals with strains. Indian mustard (Faisalabad Mustard Variety) was used as the test crop. The earthen pots were filled with 10 kg of soil that had a sandy clay loam texture. The soil had a pH of 7.64, organic matter content of 0.63%, electrical conductivity (EC) of 1.29 (dS/m), saturation percentage of 38.6%, extractable potassium concentration of 125.6 milligrams per kilogram (mg/kg), and available phosphorous concentration of 7.5 mg/kg. Lead was not detectable in the soil. Prior to the pot being filled, the soil was contaminated with lead using lead nitrate (PbNO_3_) salt as a source of lead. Subsequently, three different levels of lead contamination were established, namely, 300, 600, and 900 mg kg^−1^. The soil was allowed to reach equilibrium for a duration of two weeks after the introduction of lead contamination. The treatments were organized based on a completely randomized design, replicated thrice. Following a period of two weeks dedicated to the process of germination, thinning was conducted to preserve a single seedling within a pot. The application of NPK fertilizer in the form of Urea, DAP (Di-ammonium phosphate), and Murate of Potash was carried out using recommended doses of 145 kg ha−1 for nitrogen (N), 60 kg ha−1 for phosphorus (P), and 55 kg ha−1 for potassium (K).

One treatment was kept as control and three treatments were contaminated using lead nitrate as a source of Pb at three different levels, i.e., 300, 600, 900 mgkg^−1^, respectively. Three well-characterized bacterial strains (S2, S5, S10) were used alone as well as in each three levels of metal contamination. The strains, namely, S2 (Pseudomonas gessardii strain BLP141, Accession No. KJ547711.1), S5 (Pseudomonas fluorescens A506, Accession No. CP003041.1), and S10 (Pseudomonas fluorescens strain LMG 2189, Accession No. GU198103.1), were provided by the Soil Microbiology and Biochemistry laboratory, University of Agriculture, Faisalabad.

### 4.3. Determination of Chlorophyll a, b and Carotenoids

A fresh leaf sample of 0.5 g was thoroughly amalgamated with 10 mL acetone (80%) (*w*/*v*). The solution was filtered, and absorbance of filtrate was recorded at 663, 645, and 480 nm, respectively, for chlorophyll a, b, and carotenoids [38].

### 4.4. Determination of Antioxidant Activities and Malondialdehyde (MDA)

Ascorbate peroxidase activity was recorded following reduction in absorbance (290 nm) through ascorbate due to H_2_O_2_ [39]. Superoxidase–dismutase was recorded through reduction in superoxidenitro blue tetrazolium complex by the enzymes. Catalase activity was determined through the decomposition of H_2_O_2_ spectrophotometrically at 240 nm [40]. GR activity was recorded through the increase in absorbance (412 nm) with reduction from 5, 5 0-dithiobis (2-nitrobenzoic acid) (DTNB) to 2-nitro-5-thiobenzoic acid (TNB) [41]. For proline, the leaf was mixed with 3% sulfosalicylic acid and filtered. After adding glacial acetic acid and acid ninhydrin, the mixture was heated in a water bath and reaction was stopped after one hour using an icebox. Toluene mixture was extracted, and reading was noted at 520 nm (umol g^−1^) (Zengin and Munzuroglu, 2005). MDA was recorded through difference in absorbance (A53-A600) via Beer–Lambert’s equation (nmol g^−1^).

### 4.5. Determination of Lead in Vegetative and Reproductive Parts

Plant samples were placed in an oven at 70 °C for 24–48 h and grinded to powder-form. An amount of 0.5 g of dried grounded plant sample was placed in a flask and di-acid (HNO_3_:HCLO_4_) was added. A hot plate was used for heating the flasks until the material in the flask became clear. After cooling, all the digested material was poured into a 50 mL volumetric flask. Standard solutions were prepared for quality assurance control using lead sulphate and lead chloride, and lead contents were measured on an atomic absorption spectrophotometer (AAS) [42,43].

### 4.6. Statistical Analysis

Recorded data were analyzed statistically using computer-based statistical software (statstix 8.1). Treatment means were compared after two-way analysis of variance (ANOVA) was performed. Using the Tukey’s test/honestly significant difference (HSD) test with a 5% probability, the significant differences between the treatments were identified.

## 5. Conclusions

The presence of lead contamination had a negative impact on the growth, physiology, and yield of Indian Mustard plants. Nevertheless, the inoculation of Indian Mustard plants with Pb-tolerant rhizobacterial strains resulted in the enhancement of growth, physiology, antioxidant activities, yield, and phytoremediation potential under lead stress conditions. This study demonstrated that the application of Pb-tolerant rhizobacterial strains enhances stress tolerance in plants exposed to Pb contamination, while concurrently resulting in an increased accumulation of lead within the plant tissues. Through the implementation of this approach, the soil will undergo a process of remediation, resulting in the removal of lead contaminants. Hence, this approach showcases its potential to serve as a viable strategy for achieving effective, efficient, and cost-effective Pb bioremediation. Moreover, by utilizing the native microflora, it is possible to establish a bioremediation process for contaminated soils without causing disruption to the surrounding ecosystem.

## Figures and Tables

**Table 1:** Outcomes of lead-tolerant rhizobacterial inoculation on growth attributes of Indian mustard.

Treatment	SL (cm)	SFW (g)	SDW (g)	RL (cm)	RFW (g)	RDW (g)
Control	100.37 ± 2.1 bc	49.13 ± 1.6 b	15.02 ± 1.9 b	23.42 ± 0.5 b	18.24 ± 0.7 b	9.82 ± 1.2 b
S2	108.32 ± 3.1 a	53.37 ± 1.8 a	17.29 ± 2.1 a	26.36± 0.6 a	20.84 ± 0.4 a	11.55 ± 1.2 a
S5	107.31 ± 3.5 a	54.23 ± 2.4 a	18.19 ± 1.8 a	26.29 ± 1.6 a	20.67 ± 1.5 a	11.44 ± 0.9 a
S10	105.32 ± 1.2 ab	52.57 ± 2.9 a	17.34 ± 1.1 a	27.31± 1.8 a	20.42 ± 1.1 a	11.34 ± 0.8 a
Pb300	86.67 ± 3.2 e	43.43 ± 0.9 d	14.43 ± 0.3 cd	18.53 ± 1.8 d	14.48 ± 1.2 d	7.70 ± 0.8 de
Pb300 + S2	93.89 ± 2.8 d	47.57 ± 0.5 bc	15.23 ± 0.6 b	22.08 ± 1.7 bc	17.13 ± 1.3 bc	9.53 ± 0.7 b
Pb300 + S5	95.30 ± 1.6 cd	47.10 ± 1.9 bc	15.41 ± 0.1 b	22.56 ± 0.7 b	17.64 ± 1.9 b	9.80± 1.9 b
Pb300 + S10	92.63 ± 3.6 d	46.23 ± 1.5 c	15.09 ± 0.9 bc	22.12 ± 0.7 bc	16.91 ± 0.6 bc	9.45 ± 1.8 bc
Pb600	72.01 ± 2.1 hi	34.13 ± 2.9 h	11.43 ± 0.9 h	15.63 ± 0.8 f	12.04 ± 1.8 f	6.46 ± 1.2 f
Pb600 + S2	79.61 ± 3.8 fg	41.27 ± 1.4 de	13.51 ± 1.5 de	20.04 ± 0.7 cd	14.11 ± 1.7 de	7.81 ± 0.8 de
Pb600 + S5	83.21 ± 3.5 ef	38.37± 1.8 fg	13.12± 1.1 ef	19.52 ± 0.6 d	15.37 ± 1.9 cd	8.33 ± 0.7 d
Pb600 + S10	77.15 ± 4.1 gh	39.43± 1.2 ef	12.32 ± 1.1 eg	18.09± 1.9 d	15.01 ± 1.2 d	8.54 ± 0.4 cd
Pb900	60.12 ± 4.9 j	31.09 ± 3.7 i	9.12 ± 1.4 i	11.12 ± 1.8 g	8.96 ± 0.8 g	4.64 ± 0.4 g
Pb900 + S2	70.12 ± 4.2 i	35. 53 ± 1.6 gh	11.78 ± 0.7 gh	15.09 ± 0.5 f	12.55 ± 1.8 ef	6.40 ± 1.3 f
Pb900 + S5	69.90 ± 3.1 i	37.02 ± 1.6 fh	11.89 ± 0.9 h	16.30 ± 0.6 ef	11.52 ± 1.2 f	6.89 ± 0.6 ef
Pb900 + S10	73.45 ± 2.2 hi	35.10 ± 0.8 h	12.12 ± 0.9 fh	15.31 ± 0.7 f	11.67± 1.4 f	6.54 ± 0.8 f

Means sharing same letter(s) don’t differ significantly at *p* < 0.05.

**Table 2:** Outcomes of lead-tolerant rhizobacterial inoculation on yield and physiological attributes of Indian mustard.

Treatments	No. of Pods	No. of Seeds	Yield (g)	Chlorophyll a	Chlorophyll b	Carotenoids
	Per Plant	Per Plant	Per Plant	µg g^−1^	µg g^−1^	µg g^−1^
Control	344.37 ± 11.2 b	14.34 ± 1.9 b	18.24 ± 0.5 b	15.54 ± 0.7 b	7.11 ± 0.19 b	9.23 ± 0.7 c
S2	362.83 ± 6.5 ab	15.11 ± 1.7 ab	20.42 ± 0.8 a	16.59 ± 0.4 a	7.23 ± 0.16 a	10.34 ± 0.3 ab
S5	360.60 ± 3.4 ab	15.02 ± 2.1 ab	20.68 ± 0.7 a	16.68 ± 1.2 a	7.23 ± 0.14 a	10.56 ± 0.2 b
S10	368.49 ± 4.6 a	15.35 ± 1.3 a	20.85 ± 0.1 a	17.14 ± 1.7 a	7.18± 0.16 ab	10.78 ± 0.4 a
Pb300	292.87± 9.5 d	12.20 ± 1.1 de	14.48 ± 1.6 d	13.67 ± 1.4 d	6.09 ± 0.26 d	8.56 ± 0.6 d
Pb300 + S2	316.22 ± 10.63 c	13.17 ± 0.9 cd	16.92 ± 1.4 bc	15.04 ± 1.2 c	6.53 ± 0.12 c	9.45 ± 0.4 c
Pb300 + S5	317.31± 2.3 c	13.22 ± 0.7 cd	17.64 ± 1.1 b	15.05 ± 0.7 c	6.34 ± 0.08 c	9.78 ± 0.4 c
Pb300 + S10	322.72 ± 8.8 c	13.44 ± 0.6 bc	17.13 ± 1.5 bc	15.54 ± 0.8 bc	6.56 ± 0.27 c	9.56 ± 0.2 c
Pb600	257.90 ± 8.2 e	10.74 ± 1.0 fg	12.05 ± 2.6 f	12.09 ± 0.7 e	5.67 ± 0.16 e	7.57 ± 0.3 ef
Pb600 + S2	278.73 ± 8.7 d	11.61 ± 1.5 ef	15.00 ± 1.2 d	13.41 ± 0.4 d	5.76 ± 0.09 d	8.18 ± 0.1 de
Pb600 + S5	276.01 ± 14.6 de	11.50 ± 1.3 ef	15.38 ± 1.7 cd	13.28 ± 0.9 d	5.45 ± 0.21 de	8.21 ± 0.4 de
Pb600 + S10	279.98 ± 4.6 d	11.66 ± 1.3 ef	14.10 ± 0.8 de	13.58 ± 0.8 d	5.78 ± 0.10 d	8.48 ± 0.6 d
Pb900	205.03 ± 19.8 g	8.54 ± 0.8 h	8.97 ± 0.9 g	10.98 ± 0.7 g	4.49 ± 0.37 g	6.47 ± 0.4 h
Pb900 + S2	233.51± 8.9 f	9.72 ± 0.7 g	11.53 ± 1.5 f	11.32 ± 0.2 ef	5.10 ± 0.21 f	7.11 ± 0.6 g
Pb900 + S5	228.46 ± 4.6 f	9.51 ± 1.0 gh	12.56 ± 1.2 f	11.39 ± 0.7 e	4.89 ± 0.17 f	6.56 ± 0.4 gh
Pb900 + S10	235.81 ± 14.6 f	9.82 ± 0.5 g	11.79 ± 1.1 f	12.12 ± 0.6 ef	5.00 ± 0.18 f	7.38 ± 0.3 fg

Means sharing same letter(s) don’t differ significantly at *p* < 0.05.

**Table 3:** Outcomes of lead-tolerant rhizobacterial inoculation on antioxidant activates attributes of Indian mustard.

Treatments	APX	Catalase	MDA	GR	SOD	Proline
	µmol H_2_O_2_ mg^−1^ Protein min^−1^	µmol H_2_O_2_ mg^−1^ Protein min^−1^	nmol g^−1^	nmol NADPH mg^−1^ Protein min^−1^	mg^−1^ Protein	umol g^−1^ FW
**Control**	17.34 ± 1.9 j	348.18 ± 32.5 j	14.09± 3.5 k	154.34 ± 16.7 l	248.69 ± 30.4 j	1.21 ± 0.10 n
**S2**	19.31 ± 0.6 ij	394.40± 25.2 ij	12. 28 ± 2.4 jk	174.78 ± 12.2 kl	277.69 ± 26.5 ij	1.34 ± 0.06 lm
**S5**	21.28 ± 0.5 hi	442.23 ± 27.2 hi	11.03 ± 2.1 jk	195.79 ± 19.2 ik	311.09 ± 36.5 hi	1.56 ± 0.16 km
**S10**	20.26 ± 1.6 ij	415.56 ± 20.1 i	9.70 ± 2.6 k	184.20 ± 16.1 jk	302.87 ± 25.6 hi	1.64 ± 0.13 lm
**Pb300**	21.32 ± 0.7 hi	430.21 ± 15.6 hi	23.39 ± 3.9 eg	210.31± 22.5 hj	309.23 ± 24.6 hi	1.39 ± 0.16 jl
**Pb300 + S2**	25.11 ± 0.9 fg	531.45 ± 20.1 fg	16.10 ± 2.1 hj	241.48± 14.7 fg	374.11 ± 30.9 fg	2.11 ± 0.14 hi
**Pb300 + S5**	25.34 ± 0.7 fg	526.24 ± 12.5 g	18.39 ± 2.4 gi	233.09± 15.2 f-h	370.42 ± 28.8 fg	2.30± 0.14 hj
**Pb300 + S10**	24.01 ± 0.6 gh	494.67 ± 11.36 gh	15.22 ± 2.5 ij	219.09 ± 14.4 g-i	348.21 ± 24.1 gh	2.10 ± 0.08 ik
**Pb600**	28.21 ± 0.7 ef	597.34± 16.6 ef	33.23 ± 2.4 bc	257.10 ± 14.5 f	415.12 ± 15.9 ef	2.39 ± 0.19 gh
**Pb600 + S2**	30.47 ± 0.6 de	626.24 ± 15.6 de	28.38 ± 2.7 ce	290.21 ± 12.6 e	440.69 ± 25.4 de	2.78 ± 0.15 fg
**Pb600 + S5**	33.78 ± 1.2 c	694.65 ± 27.9 c	24.78 ± 2.0 df	307.34 ± 12.7 de	488.67 ± 20.3 cd	3.19 ± 0.21 de
**Pb600 + S10**	32.47 ± 1.7 cd	668.37 ± 29.25 cd	21.47 ± 2.7 f-h	296.10 ± 13.5 e	470.27 ± 15.5 cd	3.10 ± 0.23 ef
**Pb900**	34.39 ± 1.6 c	728.12 ± 26.5 c	47. 29 ± 3.8 a	333.32 ± 19.7 cd	500.27 ± 21.6 c	3.39 ± 0.31 cd
**Pb900 + S2**	42.12 ± 1.9 ab	889.21 ± 20.1 a	38.11 ± 4.9 b	382.65 ± 13.2 ab	625.54 ± 26.9 a	4.20 ± 0.18 ab
**Pb900 + S5**	43.21 ± 2.6 a	863.21 ± 22.3 ab	29.09 ± 2.8 cd	356.78 ± 12.5 bc	607.37 ± 27.3 ab	4.28 ± 0.29 a
**Pb900 + S10**	39.19 ± 3.7 b	805.12 ± 31.2 b	32.18 ± 4.7 c	393.19 ± 16.9 a	566.28 ± 27.9 b	3.79 ± 0.21 bc

Means sharing same letter(s) don’t differ significantly at *p* < 0.05.

## Data Availability

The data presented in this study are available on request from the corresponding author.

## References

[1] Kelley M.A., Weber D.J., Gilligan P., Cohen M.S. (2000). Breakthrough pneumococcal bacteremia in patients being treated with azithromycin and clarithromycin. Clin. Infect. Dis..

[2] Adam D. (2002). Global antibiotic resistance in *Streptococcus pneumoniae*. J. Antimicrob. Chemother..

[3] Paul M., Lador A., Grozinsky-Glasberg S., Leibovici L. (2014). Beta lactam antibiotic monotherapy versus beta lactam-aminoglycoside antibiotic combination therapy for sepsis. Cochrane Database Syst. Rev..

[4] Lis-Balchin M. (1996). Geranium oil. Int. J. Aromather..

[5] Taherpour A.A., Maroofı H., Kheradmand K. (2007). Chemical composition of the essential oil of *Pelargonium quercetorum* Agnew of *Iran*. Nat. Prod. Res..

[6] Jazayeri S.B., Amanlou A., Ghanadian N., Pasalar P., Amanlou M. (2014). A preliminary investigation of anticholinesterase activity of some Iranian medicinal plants commonly used in traditional medicine. DARU J. Pharm. Sci..

[7] Lis-Balchin M. (2002). Geranium and Pelargonium.

[8] Abouelatta A.M., Keratum A.Y., Ahmed S.I., Hisham M.E. (2020). Repellent, contact and fumigant activities of geranium (*Pelargonium graveolens* L.’Hér) essential oils against *Tribolium castaneum* (Herbst) and *Rhyzopertha dominica* (F.). Int. J. Trop. Insect Sci..

[9] Verma Ram S., Verma Sajendra K., Tandon S., Padalia Rajendra C., Darokar Mahendra P. (2020). Chemical composition and antimicrobial activity of Java citronella (*Cymbopogon winterianus* Jowitt ex Bor) essential oil extracted by different methods. J. Essent. Oil Res..

[10] Lira M.H.P.D., Andrade Júnior F.P.D., Moraes G.F.Q., Macena G.D.S., Pereira F.D.O., Lima I.O. (2020). Antimicrobial activity of geraniol: An integrative review. J. Essent. Oil Res..

[11] Baliyan S., Mukherjee R., Priyadarshini A., Vibhuti A., Gupta A., Pandey R.P., Chang C.M. (2022). Determination of antioxidants by DPPH radical scavenging activity and quantitative phytochemical analysis of *Ficus religiosa*. Molecules.

[12] Tepe B., Sokmen M., Akpulat H.A., Yumrutas O., Sokmen A. (2006). Screening of antioxidative properties of the methanolic extracts of *Pelargonium endlicherianum* Fenzl., *Verbascum wiedemannianum* Fisch. & Mey., *Sideritis libanotica* Labill. subsp. *linearis* (Bentham) Borm. *Centaurea mucronifera* DC. and *Hieracium cappadocicum* Freyn. from Turkish flora. Food Chem..

[13] Şeker Karatoprak G., Göger F., Yerer M.B., Koşar M. (2017). Chemical composition and biological investigation of *Pelargonium endlicherianum* root extracts. Pharm. Biol..

[14] Boukhris M., Simmonds M.S., Sayadi S., Bouaziz M. (2013). Chemical composition and biological activities of polar extracts and essential oil of rose-scented geranium, *Pelargonium graveolens*. Phytother. Res..

[15] Luqman S., Dwivedi G.R., Darokar M.P., Kalra A., Khanuja S.P. (2007). Potential of rosemary oil to be used in drug-resistant infections. Altern. Ther. Health Med..

[16] Su J.Y., Zhu L., Tian Y.J. (2012). Chemical composition and antimicrobial activities of essential oil of *Matricaria songarica*. Int. J. Agric. Biol..

[17] Hemaiswarya S., Doble M. (2009). Synergistic interaction of eugenol with antibiotics against Gram negative bacteria. Phytomedicine.

[18] Lan A., Xu W., Zhang H., Hua H., Zheng D., Guo R., Shen N., Hu F., Feng J., Liu D. (2013). Inhibition of ROS-activated p38MAPK pathway is involved in the protective effect of H2S against chemical hypoxia-induced inflammation in PC12 cells. Neurochem. Res..

[19] Gibbons S., Oluwatuyi M., Veitch N.C., Gray A.I. (2003). Bacterial resistance modifying agents from *Lycopus europaeus*. Phytochemistry.

[20] Kim J., Jayaprakasha G.K., Uckoo R.M., Patil B.S. (2012). Evaluation of chemopreventive and cytotoxic effects of lemon seed extracts on human breast cancer (MCF-7) cells. Food Chem. Toxicol..

[21] Teale C.J. (2002). Antimicrobial resistance and the food chain. J. Appl. Microbiol..

[22] Choi S.H., Lim S., Shin S.W. (2007). Combined effects of the essential oil from *Pelargonium graveolens* with antibiotics against *Streptococcus pneumoniae*. Nat. Prod. Res..

[23] McLafferty F.W., Stauffer D.B. (1989). The Wiley/NBS Registry of Mass Spectral Data.

[24] Hochmuth D.H. (2008). MassFinder 4.0.

[25] Gyamfi M.A., Yonamine M., Aniya Y. (1999). Free-radical scavenging action of medicinal herbs from Ghana Thonningia Sanguinea on experimentally induced liver injuries. Gen. Pharmacol..

[26] Oomah B.D., Mazza G. (1996). Flavonoids and antioxidative activities in buckwheat. J. Agric. Food Chem..

[27] Re R., Pellegrini N., Proteggente A., Pannala A., Yang M., Rice-Evans C. (1999). Antioxidant activity applying an improved ABTS radical cation decolorisation assay. Free Radic. Bio Med..

[28] Yin X., Knecht D.A., Lynes M.A. (2005). Metallothionein mediates leukocyte chemotaxis. BMC Immunol..

[29] Köngül Şafak E., Şeker Karatoprak G., Dirmenci T., Duman H., Küçükboyacı N. (2022). Cytotoxic effects of some Nepeta species against breast cancer cell lines and their associated phytochemical properties. Plants.

[30] Yap P.S.X., Krishnan T., Chan K.G., Lim S.H.E. (2015). Antibacterial mode of action of *Cinnamomum verum* bark essential pil, alone and in combination with piperacillin, against a multi-drug-resistant *Escherichia coli* strain. J. Microbiol. Biotechnol..

[31] CLSI M100-Performance Standards of Antimicrobial Susceptibility Testing.

[32] (2018). European Committee on Antimicrobial Susceptibility Testing (EUCAST), Breakpoint Tables for Interpretation of MICs and Zone Diameters, Vol. 8, EUCAST, Vaxjo, Sweden. https://www.eucast.org/clinical_breakpoints.

[33] Dumlupinar B., Karatoprak G.Ş., Celik D.D., Gürer Ü.S., Demirci B., Gürbüz B., Rayaman P., Kurtulus E.M. (2020). Synergic potential of *Pelargonium endlicherianum* Fenzl. essential oil and antibiotic combinations against *Klebsiella pneumoniae*. S. Afr. J. Bot..

[34] Dumlupinar B., Celik D.D., Karatoprak G.Ş., Gürer Ü.S. (2022). Synergy between *Pelargonium endlicherianum* essential oil and conventional antibiotics against *Neisseria meningitidis* and *Haemophilus influenzae*. S. Afr. J. Bot..

[35] Lategan K., Fowler J., Bayati M., Fidalgo de Cortalezzi M., Pool E. (2018). The effects of carbon dots on immune system biomarkers, using the murine macrophage cell line RAW 264.7 and human whole blood cell cultures. Nanomaterials.

[36] Pruul H., Mcdonald P.J. (1979). Enhancement of leukocyte activity against *Escherichia coli* after brief exposure to chloramphenicol. Antimicrob. Agents Chemother..

[37] Novelli A., Fallani S., Cassetta M.I., Conti S., Mazzei T. (2000). Postantibiotic leukocyte enhancement of meropenem against gram-positive and gram-negative strains. Antimicrob. Agents Chemother..

[38] Ćavar S., Maksimović M. (2012). Antioxidant activity of essential oil and aqueous extract of *Pelargonium graveolens* L’Her. Food Control.

[39] Braca A., Politi M., Sanogo R., Sanou H., Morelli I., Pizza C., De Tommasi N. (2003). Chemical composition and antioxidant activity of phenolic compounds from wild and cultivated *Sclerocarya birrea* (Anacardiaceae) leaves. J. Agric. Food Chem..

[40] Liyana-Pathirana C.M., Shahidi F. (2006). Antioxidant properties of commercial soft and hard winter wheats (*Triticum aestivum* L.) and their milling fractions. J. Sci. Food Agric..

[41] Gaamoune S., Harzallah D., Kada S., Dahamna S. (2014). The comparison of two tannin extraction methods from *Galium tunetanum* Poiret. and their antioxidant capacities. Der Pharm. Lett..

[42] Petlevski R., Flajs D., Kalođera Z., Končić M.Z. (2013). Composition and antioxidant activity of aqueous and ethanolic *Pelargonium radula* extracts. S. Afr. J. Bot..

[43] Prior R.L., Cao G. (1999). In vivo total antioxidant aapacity: Comparison of different analytical methods. Free Radic. Bio. Med..

